# The role of the urologist, BCG vaccine administration, and SARS‐CoV‐2: An overview

**DOI:** 10.1002/bco2.21

**Published:** 2020-06-22

**Authors:** Nathan A. Brooks, Vikram Narayan, Paul K. Hegarty, Helen Zafirakis, Xiang‐Yang Han, Ashish M. Kamat

**Affiliations:** ^1^ Department of Urology The University of Texas MD Anderson Cancer Center Houston TX USA; ^2^ Department of Urology Mater Private Hospital Dublin Ireland; ^3^ Department of Pathology and Laboratory Medicine MD Anderson Cancer Center The University of Texas Houston TX USA

**Keywords:** bacillus Calmette-Guerin, BCG, COVID-19, SARS-CoV-2

## Abstract

**Objectives:**

To summarize the available literature regarding bacillus Calmette‐Guerin (BCG) administration, severe acute respiratory syndrome conoravirus‐2 (SARS‐CoV‐2), and the resulting clinical condition coronavirus disease (COVID‐19) in light of recent epidemiologic work suggesting decreased infection severity in BCG immunized populations while highlighting the potential role of the urologist in clinical trials and ongoing research efforts.

**Materials and methods:**

We reviewed the available literature regarding COVID‐19 and BCG vaccination. Specifically, the epidemiologic evidence for decreased COVID‐19 morbidity in countries with BCG vaccination programs, current clinical trials for BCG vaccination to protect against COVID‐19, potential mechanisms and rationale for this protection, and the role of the urologist and urology clinic in providing support and/or leading ongoing efforts.

**Results:**

Epidemiologic evidence suggests that the crude case fatality rates are lower for countries with BCG vaccination compared to those without such programs. Four prospective, randomized clinical trials for BCG vaccination were identified including NCT04348370 (BADAS), NCT04327206 (BRACE), NCT04328441 (BCG‐CORONA), and NCT04350931. BCG administration may contribute to innate and adaptive immune priming with several opportunities for translational research.

**Conclusions:**

The urologist’s expertise with BCG and the infrastructure of urologic clinics may afford several opportunities for collaboration and leadership to evaluate and understand the potential role of BCG in the current COVID‐19 pandemic.

## 1 INTRODUCTION

Infections with the severe acute respiratory syndrome coronavirus‐2 (SARS‐CoV‐2) and the resulting clinical condition coronavirus disease (COVID‐19) have resulted in a worldwide pandemic with nearly 3.8 million confirmed infections worldwide and 270 000 deaths as of May 8, 2020.^1^ These numbers are expected to continue to grow, though at differing rates and patterns depending on the mitigation strategies employed.^2^ Viral RNA shedding may continue after recovery from disease leading to issues with possible reinfection or, more likely, residual infection.^3^ Not only has the morbidity of this disease stressed patients, families, and underprepared healthcare systems, but it also has the potential to enact long‐term, fundamental change in daily life for billions of people.^4^ The observed morbidity of COVID‐19 will be further magnified in vulnerable populations: namely the elderly, patients with cancer undergoing treatment, patients of low socioeconomic status with poor access to care and chronic conditions, as well as in countries in which health system preparedness is lacking and where the economic impacts might be devasting.^5^, ^6^, ^7^, ^8^

Successful management of this pandemic requires: containment, therapies to reduce clinical morbidity and mortality, and ultimately, vaccination to prevent infection. Currently, multiple therapeutic agents are being evaluated to lessen the clinical morbidity of COVID‐19 with varying degrees of success.^9^ These include anti‐malarial medications,^10^ antiviral medications,^11^ and convalescent serum harvested from patients recovered from the infection.^12^ The rational development of a vaccine is of paramount importance.^13^ New vaccine development, however, will take time, trial, and error and must be developed and distributed on a global scale.^14^, ^15^, ^16^ The bacillus Calmette‐Guerin (BCG) vaccine has been administered for the prevention of tuberculosis (TB) in many countries worldwide to almost four billion persons since its first human use was described in 1921.^17^ The BCG vaccine provides protection from TB infection in 40‐60% of recipients and has demonstrated relative safety with a rate of serious adverse events approaching zero.^18^ In both animal and human studies, BCG vaccination provides a non‐specific benefit to the immune system, relative protection against, and reduced mortality from infections by other microbes (bacteria and viruses) which may occur by epigenetic reprogramming and induction of trained immunity.^19^, ^20^

Bacillus Calmette‐Guerin is a well‐known medication to urologists as it is administered intravesically as therapy for many patients with non‐muscle invasive bladder cancer (NMIBC). Emerging evidence suggests that BCG vaccination might reduce the rate of infection and mitigate the rate of mortality in countries with BCG vaccination programs.^21^ Herein, we review the current literature regarding BCG vaccination and COVID‐19, currently enrolling clinical trials, and the potential to leverage the expertise of the urologist and the urology clinic in supporting these trials.

## 2 ROLE OF BCG IN NON‐MUSCLE INVASIVE BLADDER CANCER

Clinical trials have shown that BCG immunotherapy prevents cancer recurrence, progression, reduces the need for cystectomy, and prolongs survival for patients with NMIBC.^22^ For intravesical therapy, the schedule of BCG treatment comprises an induction course (6 weekly treatments) and a maintenance courses of three instillations spaced one week apart, at 3 and 6 months after induction and then every 6 months thereafter, for a total of seven sets of maintenance. Thus, a complete treatment of induction and maintenance adds up to 27 instillations over 3 years.^23^ Since BCG is a live, attenuated mycobacterium, it must be handled appropriately. Urologic clinics, pharmacists, and nurses are familiar with the safe preparation, handling, and disposal of BCG.

Over 95% of treated patients have no significant toxicity, however, serious and even fatal toxicity can occur if BCG is not administered appropriately. Because of the sheer number of installations per patients, urologists are experienced in caring for patients with complications of BCG instillation and, should such complications occur with BCG vaccination, may be uniquely positioned to manage such complications. The SWOG PRIME trial evaluating priming patients with subcutaneous BCG 3 weeks prior to intravesical BCG is ongoing. This trial was somewhat prescient since, while urologists do not routinely perform BCG vaccination, trial sites will have additional expertise with vaccine administration.^24^

There are reasonable concerns about allocation of BCG for COVID‐19 given the shortages of both the intravesical and vaccination formulations worldwide.^25^, ^26^ When Kamat and colleagues were designing the BADAS study in early March, they reached out to the bladder cancer patient community and Bladder Cancer Advocacy Network (BCAN), for input and support for diversion of some of the drug (BCG) to the study. As always, the patients and BCAN were altruistic and supportive.

Additionally, some consider BCG as a class I‐II pathogen and it is recommended that it be reconstituted in a certified biological cabinet and used within 2 hours per the manufacturer’s label.^26^ Recently, we presented evidence for TICE® BCG (Merck, Kenilworth, NJ) that BCG organisms remain viable for at least 8 hours after reconstitution when stored on ice and in the absence of light.^27^ Additional support for a longer duration of viability was demonstrated for at least 4 hours,^28^ and guidance from the World Health Organization (WHO) on administration of BCG vaccination for TB suggests that the vaccine may be administered up to 6 hours after reconstitution.^29^ Such considerations are important as one vial of BCG is used for one patient with bladder cancer but can be used to inoculate 500 individuals for vaccination (which is 1/500th the dose, based on CFU). In order to deliver 500 doses from one vial within 2 hours, 4.2 vaccines would need to be administered every minute. For clinical trials, extending BCG reconstitution time may be important to ensure that vaccine is not wasted in times of shortage for either bladder cancer patients or TB vaccination.^30^

## 3 THE LINK BETWEEN BCG, SYSTEMIC DISEASE REDUCTION, AND SARS‐CoV‐2

Members of our group recently also described differences in the observed crude case fatality rates (CFR) of COVID‐19 between countries with active BCG vaccination programs for tuberculosis when compared to those that do not routinely vaccinate.^21^ Starting from data extrapolated from March 22, 2020, the daily incidence of COVID‐19 was 0.8/million in countries with a BCG vaccination program compared to nearly 34.8/million in countries without such a program (Table 1). There was considerable observational overlap between the countries without an active BCG vaccination program and those that are most affected by COVID‐19 in Europe, with CFR estimates around 4.1% in countries with a BCG vaccination program compared to 5.1% in those without. These analyses may in part be explained by numerous confounding factors, including heterogeneity, likely lower testing rates within these countries, the lack of a confirmation of true BCG vaccination status among those affected, and an underestimation of asymptomatic cases, but highlight the need for further study. The World Health Organization (WHO) acknowledges the potential bias of the aforementioned ecological study and recommends studying the potential impact of BCG on COVID‐19 in clinical trials, such as the BADAS trial (www.bcgbadas.org), rather than simply recommending BCG vaccination to all, as is being done in some countries.^31^ Given BCG supply shortages for both intravesical and vaccine formulations, the WHO additionally cautions that overuse of the BCG vaccine for COVID‐19 prevention without additional, prospective data may prove harmful.

**Table 1:** The daily incidence of COVID‐19 cases and mortality rate in European Countries on March 22, 2020. Adapted from Hegarty et al^21^

BCG vaccination program	Daily incidence (per million)	Mortality rate (per million)
Current	0.8	0.08
None	34.8	34.8

Evidence supports the immunomodulatory potential of BCG administration. Neonatal BCG vaccination has been associated with a 50% reduction in neonatal mortality in developing nations, with vaccinated infants having fewer cases of respiratory infections and sepsis.^32^ Arts and colleagues were able to demonstrate additional heterologous benefits of BCG vaccination by performing a randomized controlled study in which 30 healthy Dutch men received either placebo or BCG (Denmark strain), following which all participants received yellow fever vaccination after 30 days.^19^ Participants who had received BCG vaccination were found to have a reduction in yellow fever viremia. Furthermore, in this study, when the participants were BCG vaccinated and then later challenged with yellow fever virus, they had decreased “cytokine storm,” —IFNa, IFNg, IL1Ra, IL8, and TNF all decreased.^19^ This would be expected because it is well known that BCG stimulates a TH1 response; whereas, yellow fever (and most likely COVID‐19) stimulates at TH2 response. Tipping the response one way or the other usually mitigates the other response, so it is not surprising that a TH1 stimulant reduces a TH2 response. This is particularly of interest as decreased levels of cytokines might result in lessened disease severitywithout impacting longer term humoral antibody.

Bacillus Calmette‐Guerin vaccination priming has also been evaluated in conjunction with the seasonal influenza vaccine. Forty individuals were randomized to receive either placebo or BCG vaccination after which all participants were given an intramuscular injection of the 2013‐2014 seasonal influenza vaccine (A[H1N1]pdm09, A[H3N2]2012, and B/2012). BCG‐vaccinated subjects were found to have enhanced antibody responses to the A(H1N1)pdm09 vaccine strain compared to placebo‐treated participants, with increased production of IFN‐y and IL‐6.^33^ The reasons for this variability in response mechanisms governing these findings are not known.

Informed in part by these data, there are currently four randomized controlled trials looking to investigate what impact, if any, BCG vaccination can have in reducing COVID‐19 morbidity (Table 2). The Reducing HealthCare Workers Absenteeism in COVID‐19 Pandemic Through BCG Vaccine (BCG‐CORONA) trial (NCT04328441), led by a Dutch research group, is a multicenter RCT in which healthcare workers with direct patient contact to COVID‐19 patients are randomized to receive either BCG vaccine (Danish strain) or placebo. The primary outcome of this trial, which aims to enroll 1500 participants, is healthcare worker absenteeism, defined as the number of days of unplanned absenteeism for any reason. Secondary measures include a variety of SARS‐CoV‐2–related metrics, including cumulative incidence of infection, days of self‐reported fever or respiratory symptoms, hospital admissions related to COVID‐19, and other endpoints. In Australia, researchers have opened the** **BCG vaccination to Protect Healthcare Workers Against COVID‐19 (BRACE) trial** **(NCT04327206), a similar project focused on enrolling 4000 healthcare workers. Participants will be randomized to receive either BCG vaccine (Danish strain) or no vaccination and followed for 12 months. The co‐primary endpoints are the incidence of COVID‐19, as defined by fever, at least one sign or symptom of respiratory disease, plus a positive SARS‐CoV‐2 test as well as the incidence of severe COVID‐19 disease, defined by the number of participants who were either admitted to the hospital or died in the context of a positive SARS‐CoV‐2 test.

**Table 2:** Ongoing trials investigating BCG vaccination as a mitigation factor against COVID‐19 infection/morbidity

Study^a^	Design	Country	Population	BCG strain
NCT04327206 (BRACE)	Natural‐history RCT	Australia	Healthcare workers	Danish strain
NCT04328441 (BCG‐CORONA)	Placebo‐controlled RCT	Netherlands	Healthcare workers	Danish strain
NCT04348370 (BADAS)	Placebo‐controlled RCT	United States	Healthcare workers	TICE
NCT04350931	Placebo‐controlled RCT	Egypt	Healthcare workers	Danish strain

Abbreviation: RCT, randomized controlled trial.

^a^ Clinicaltrials.gov registration number.

In the United States, a multicenter trial‐BCG Vaccine for HealthCare Workers as Defense Against SARS‐CoV‐2 (BADAS; PI: Kamat, DiNardo, Murray) (NCT04348370) aims to randomize an initial group of 700 hospital personnel at three sites, to be expanded nationwide, who are taking care of patients with known or suspected SARS‐CoV‐2 infection to receive either BCG (TICE strain) or placebo. The primary endpoint of this study is incidence of SARS‐CoV‐2 (measured by a positive test) with secondary endpoints to include disease‐related severity (admission to the hospital, oxygen requirement, treatment in intensive care, ventilator requirement, and death). A similar study involving 900 healthcare workers is also planned in Egypt, with use of the Danish BCG strain vaccine (NCT04350931). The ultimate goal of all these studies is to inform the decision to use BCG as a vaccine for all individuals at high risk of exposure, for example, healthcare workers, law enforcement, food delivery and supply workers, the elderly, nursing home residents etc. Indeed, this is being proposed in many parts of the world already; however, we believe that sound studies are needed before such blanket recommendations can be made.

## 4 IMMUNOLOGICAL INTERPLAY: BCG, BLADDER CANCER, AND SARS‐CoV

Virus‐specific memory T cells are crucial in broad and long‐term protection against SARS‐CoV infection^34^ and this has been clearly documented in several animal models.^35^ Structural antigens of SARS‐CoV act as a major antigen for both humoral and cellular immunity.^36^ SARS‐CoV‐specific T cells are important for the recognition and clearance of infected cells, particularly in the lungs of SARS‐CoV infected individuals^37^ and cytotoxic T cells are central to this mechanism.^38^

The anti‐tumor effect of BCG involves a complex interplay between the direct effect of BCG on the tumor and stimulation of the immune response allowing for recognition of tumor antigens via long‐term, adaptive immunity.^39^, ^40^ Potential mechanisms of action have been described by Zlotta et al^41^ and Redelman‐Sidi et al^42^ BCG binds to urothelial cells through fibronectin and integrins. BCG is internalized into urothelial cells and encounters the first line of innate immune response cells. Major histocompatibility complex (MHC) II is upregulated and IL‐6, IL ‐8, and granulocyte‐macrophage colony‐stimulating factor (GM‐CSF) are produced leading to recruitment of immune cells. These cells are made up of granulocytes, CD4 and CD8 T cells, natural killer (NK) cells, and macrophages. Immunologic cells produce a wave of mainly TH‐1 cytokines, that is, Interferon gamma, IL‐1, IL‐12, IL‐18, IL‐23, and tumor necrosis factor (TNF)‐alpha. Induction of Th2‐cells via NK cells (driven by IL‐4, IL‐5, IL‐6, and IL‐10), neutrophil recruitment (via IL‐17 release), and macrophage provides innate immunity. BCG vaccination additionally confers epigenetic changes to human monocytes, with vaccinated individuals demonstrating increased histone H3 acetylation at lysine 27 (H3K27ac) at 30 days with the most pronounced changes observed in signaling pathways and genes involved in modulating inflammatory response and cytokine production.^43^ BCG activity in the bladder, closely mirrors the action of BCG when administered sub‐dermally as its protective effect against mycoplasma and other infections. Thus, evidence suggests a potential mechanistic link between immunomodulation and priming when BCG administration occurs via any route. Furthermore, BCG induces a Th1 response while SARS‐CoV‐2 (and other viruses) elicit predominantly a Th2 response. These studies, thus, suggest a potential mechanistic rationale for protective effect of BCG against the cytokine storm induced by COVID‐19 infections, which will be studied in the ongoing trials.

Bacillus Calmette‐Guerin is currently administered by several routes including: intravesically, sub‐dermally, directly injected into certain tumors, intra‐nasally, pharyngeally or as an inhalation spray directly into the lungs. There presents an opportunity to re‐purpose intravesical BCG for use as a potential protective agent against SARS‐Cov‐2. In addition to consideration for intradermal injection, inhalation or nebulization may be considered as, similarly to the bladder, this may allow BCG to act directly the upper and lower respiratory system affected by SARS‐Cov‐2. A potential inhalational form of BCG has already been developed and BCG may induce pulmonary mucosal immune responses at the point of entry of the pathogen.^44^ The safety of the aerosolized BCG vaccine is well established without reported side effects.^45^

## 5 BCG VACCINATION IN THE LABORATORY

Many opportunities exist for the urologist and urology infrastructure to contribute to translational and basic scientific correlates to further the understanding of BCG vaccination in preventing COVID‐19. Several animal models focusing on SARS‐CoV‐1 have previously been described. Viral replication has been reported in mice, hamsters, cats, civets, and primates, however, recreation of human disease in these models without viral passage to produce animal‐adapted variants has proven difficult.^46^ For SARS‐CoV‐2, research efforts have identified that viral replication occurs in the respiratory tract of ferrets and cats when directly inoculated with virus.^47^ Infection and rapid transmission of SARS‐CoV‐2 has been demonstrated in ferrets.^48^ Utilizing BCG vaccination in emerging animal models may help to better understand the rate of generation of immunity, the mechanism of possible immunity and/or disease severity reduction, and the optimal dose of BCG to administer^49^ on a timescale allowing for more rapid understanding and deployment with the caveat that animals models do not fully recapitulate human disease pathogenesis.^50^ For translational efforts, the urologist and urology facilities may play a pivotal role in BCG reconstitution and administration including opportunities to play a pivotal role on, or in leading the research team.

## 6 CONCLUSIONS

The administration of the BCG vaccine, both for bladder cancer and for the prevention of TB, is safe and has been utilized in over four billion people. There is an association between BCG vaccination and decreased childhood lung‐related mortality as well as improved vaccination efficacy for yellow fever and some influenza strains. While the exact mechanism of action is non‐specific, improved innate immune priming, T‐cell polarization, and epigenetic changes play a role in the activity of BCG. Epidemiologic evidence has demonstrated a possible association between BCG vaccination rates and crude case fatality rates from COVID‐19. Caution must be urged in the interpretation of this data to ensure BCG stewardship, as association does not represent causality. However, taken in aggregate, all these data have prompted four clinical trials of BCG vaccination in healthcare workers worldwide. Because of our familiarity with BCG, urologists and our teams are uniquely equipped to contribute and/or lead ongoing clinical and translational research efforts as the world seeks to dampen the global crisis caused by COVID‐19.

## CONFLICT OF INTEREST

NAB: Dr. Brooks has nothing to disclose; VN: Dr. Narayan has nothing to disclose; PKH: Dr. Hegarty has nothing to disclose; HZ: Dr. Zafirakis has nothing to disclose; ZYH: Dr. Han has nothing to disclose; AMK: Dr. Kamat reports personal fees and other from Merck, BMS, Eisai, Photocure, other from Arquer, MDx Health, FKD Industries, personal fees from Astra Zeneca, IBCG, TMC Innovation, Theralase, BioClin Therapeutics, Cepheid, Medac, Asieris, Pfizer, Abbott Molecular, US Biotest, Ferring, Imagin, Cold Genesys, Roviant, Sessen Bio, Nucleix, enGene, ArTara, Janssen, Seattle Genetics, and grants from CEC Oncology, outside the submitted work; In addition, Dr. Kamat has a patent CyPRIT‐Cytokine Panel for Response to Intravesical Immunotherapy pending.

## References

[1] Dong E , Du H , Gardner L . An interactive web‐based dashboard to track COVID‐19 in real time. Lancet Infect Dis;20(5):533‐34. 32087114 10.1016/S1473-3099(20)30120-1 PMC7159018

[2] Anderson RM , Heesterbeek H , Klinkenberg D , Hollingsworth TD . How will country‐based mitigation measures influence the course of the COVID‐19 epidemic? Lancet. 2020;395(10228):931–4. 32164834 10.1016/S0140-6736(20)30567-5 PMC7158572

[3] Wang M , Hu Z , Liu J , Pang P , Fu G , Qian A , et al. Positive RT‐PCR test results in discharged COVID‐19 patients. Reinfection or Residual? 2020. 10.21203/rs.3.rs-18042/v1

[4] Wang H‐y , Xia Q , Xiong Z‐z , Li Z‐x , Xiang W‐y , Yuan Y‐w , et al. The psychological distress and coping styles in the early stages of the 2019 coronavirus disease (COVID‐19) epidemic in the general mainland Chinese population: a web‐based survey. medRxiv. 2020;15(5):e0233410. 10.1371/journal.pone.0233410 PMC7224553 32407409

[5] Gilbert M , Pullano G , Pinotti F , Valdano E , Poletto C , Boëlle P‐Y , et al. Preparedness and vulnerability of African countries against importations of COVID‐19: a modelling study. Lancet. 2020;395(10227):871–7. 32087820 10.1016/S0140-6736(20)30411-6 PMC7159277

[6] Lloyd‐Sherlock P , Ebrahim S , Geffen L , McKee M . Bearing the brunt of covid‐19: older people in low and middle income countries. BMJ. 2020;368:m1052. 32169830 10.1136/bmj.m1052

[7] McKibbin WJ , Fernando R . The global macroeconomic impacts of COVID‐19: seven scenarios. SSRN Electronic J. 2020. 10.2139/ssrn.3547729

[8] Liang W , Guan W , Chen R , Wang W , Li J , Xu K , et al. Cancer patients in SARS‐CoV‐2 infection: a nationwide analysis in China. Lancet Oncol. 2020;21(3):335–7. 32066541 10.1016/S1470-2045(20)30096-6 PMC7159000

[9] Liu C , Zhou Q , Li Y , Garner LV , Watkins SP , Carter LJ , et al. Research and development on therapeutic agents and vaccines for COVID‐19 and related Human Coronavirus Diseases. ACS Cent Sci. 2020;6(3):315–31. 32226821 10.1021/acscentsci.0c00272 PMC10467574

[10] Cortegiani A , Ingoglia G , Ippolito M , Giarratano A , Einav S . A systematic review on the efficacy and safety of chloroquine for the treatment of COVID‐19. J Crit Care. 2020;57:279–83. 32173110 10.1016/j.jcrc.2020.03.005 PMC7270792

[11] Cao B , Wang Y , Wen D , Liu W , Wang J , Fan G , et al. A trial of Lopinavir‐Ritonavir in adults hospitalized with severe Covid‐1. N Engl J Med. 2020;382:1787‐99. 32187464 10.1056/NEJMoa2001282 PMC7121492

[12] Casadevall A , Pirofski L‐a . The convalescent sera option for containing COVID‐19. J Clin Investig. 2020;130(4):1545–8. 32167489 10.1172/JCI138003 PMC7108922

[13] Peeples L . News feature: avoiding pitfalls in the pursuit of a COVID‐19 vaccine. Proc Natl Acad Sci. 2020;117(15):8218–21. 32229574 10.1073/pnas.2005456117 PMC7165470

[14] Lurie N , Saville M , Hatchett R , Halton J . Developing covid‐19 vaccines at pandemic speed. N Engl J Med. 2020;382(21):1969–73. 32227757 10.1056/NEJMp2005630

[15] Prompetchara E , Ketloy C , Palaga T . Immune responses in COVID‐19 and potential vaccines: lessons learned from SARS and MERS epidemic. Asian Pac J Allergy Immunol. 2020;38:1–9. 32105090 10.12932/AP-200220-0772

[16] Yamey G , Schäferhoff M , Hatchett R , Pate M , Zhao F , McDade KK . Ensuring global access to COVID‐19 vaccines. Lancet. 2020;395:1405–6. 32243778 10.1016/S0140-6736(20)30763-7 PMC7271264

[17] Oettinger T , Jørgensen M , Ladefoged A , Hasløv K , Andersen P . Development of the Mycobacterium bovis BCG vaccine: review of the historical and biochemical evidence for a genealogical tree. Tuber Lung Dis. 1999;79(4):243–50. 10692993 10.1054/tuld.1999.0206

[18] Roy A , Eisenhut M , Harris RJ , Rodrigues LC , Sridhar S , Habermann S , et al. Effect of BCG vaccination against *Mycobacterium tuberculosis* infection in children: systematic review and meta‐analysis. BMJ. 2014;349:g4643. 25097193 10.1136/bmj.g4643 PMC4122754

[19] Arts RJW , Moorlag SJCFM , Novakovic B , Li Y , Wang S‐Y , Oosting M , et al. BCG vaccination protects against experimental viral infection in humans through the induction of cytokines associated with trained immunity. Cell Host Microbe. 2018;23(1):89–100.e5. 29324233 10.1016/j.chom.2017.12.010

[20] Kleinnijenhuis J , Quintin J , Preijers F , Benn CS , Joosten LAB , Jacobs C , et al. Long‐lasting effects of BCG vaccination on both heterologous Th1/Th17 responses and innate trained immunity. J Innate Immun. 2014;6(2):152–8. 24192057 10.1159/000355628 PMC3944069

[21] Hegarty PK , Sfakianos JP , Giannarini G , DiNardo AR , Kamat AM . COVID‐19 and bacillus Calmette‐Guérin: What is the link? European Urology. Oncology. 2020. 10.1016/j.euo.2020.04.001 PMC7152883 32327396

[22] Sylvester RJ , van der Meijden A , Lamm Dl . Intravesical bacillus Calmette‐Guerin reduces the risk of progression in patients with superficial bladder cancer: a meta‐analysis of the published results of randomized clinical trials. J Urol. 2002;168(5):1964–70. 12394686 10.1016/S0022-5347(05)64273-5

[23] Lamm D . Optimal BCG treatment of superficial bladder cancer as defined by American trials. Eur Urol. 1992;21:12–6. 1396941 10.1159/000474915

[24] Svatek RS , Tangen C , Delacroix S , Lowrance W , Lerner SP . Background and update for S1602 “A Phase III randomized trial to evaluate the influence of BCG strain differences and T cell priming with intradermal BCG before intravesical therapy for BCG‐naïve high‐grade non‐muscle‐invasive bladder cancer. Eur Urol Focus. 2018;4(4):522–4. 30197040 10.1016/j.euf.2018.08.015 PMC6581029

[25] Hegarty PK , Sfakianos JP , Giannarini G , DiNardo AR , Kamat AM . COVID‐19 and Bacillus Calmette‐Guérin: What is the link? Eur Urol Oncol. 2020;S2588–9311(20):30049–3. 10.1016/j.euo.2020.04.001 PMC7152883 32327396

[26] Bandari J , Maganty A , MacLeod LC , Davies BJ . Manufacturing and the market: rationalizing the shortage of Bacillus Calmette‐Guérin. Eur Urol Focus. 2018;4(4):481–4. 30005997 10.1016/j.euf.2018.06.018

[27] Brooks N , Nagaraju S , Matulay JT , Han X‐Y , Kamat AM . BCG shortage: reassessing the clinical viability of Bacillus Calmette‐Guerin (BCG) after reconstitution. American Society of. J Clin Oncol. 2020;38(6_suppl):534.

[28] Conly J , Phillips A , Campbell I , Dedier H , Cork L . Stability of reconstituted freeze‐dried Bacille Calmette‐Guérin used for intravesical immunotherapy for bladder cancer. Can J Urol. 1998;5(4):608–10. 11305964

[29] World Health Organization . Temperature sensitivity of vaccines. Geneva, Switzerland:World Health Organization; 2006.

[30] Harris RC , Dodd PJ , White RG . The potential impact of BCG vaccine supply shortages on global paediatric tuberculosis mortality. BMC Med. 2016;14(1):138. 27633883 10.1186/s12916-016-0685-4 PMC5025545

[31] World Health Organization . Bacille Calmette‐Guérin (BCG) vaccination and COVID‐19: scientific brief, 12 April 2020. World Health Organization; 2020.

[32] Aaby P , Roth A , Ravn H , Napirna BM , Rodrigues A , Lisse IM , et al. Randomized trial of BCG vaccination at birth to low‐birth‐weght cildren: beneficial nnspecific efects in the nonatal priod? J Infect Dis. 2011;204(2):245–52. 21673035 10.1093/infdis/jir240

[33] Leentjens J , Kox M , Stokman R , Gerretsen J , Diavatopoulos DA , van Crevel R , et al. BCG vaccination enhances the immunogenicity of subsequent influenza vaccination in healthy volunteers: a randomized, placebo‐controlled pilot study. J Infect Dis. 2015;212(12):1930–8. 26071565 10.1093/infdis/jiv332

[34] Channappanavar R , Fett C , Zhao J , Meyerholz DK , Perlman S . Virus‐specific memory CD8 T cells provide substantial protection from lethal severe acute respiratory syndrome coronavirus infection. J Virol. 2014;88(19):11034–44. 25056892 10.1128/JVI.01505-14 PMC4178831

[35] Takano T , Morioka H , Gomi K , Tomizawa K , Doki T , Hohdatsu T . Screening and identification of T helper 1 and linear immunodominant antibody‐binding epitopes in spike 1 domain and membrane protein of feline infectious peritonitis virus. Vaccine. 2014;32(16):1834–40. 24530149 10.1016/j.vaccine.2014.01.074 PMC7115422

[36] Du L , Zhao G , Chan CC , Sun S , Chen M , Liu Z , et al. Recombinant receptor‐binding domain of SARS‐CoV spike protein expressed in mammalian, insect and *E. coli* cells elicits potent neutralizing antibody and protective immunity. Virology. 2009;393(1):144–50. 19683779 10.1016/j.virol.2009.07.018 PMC2753736

[37] Gu J , Gong E , Zhang B , Zheng J , Gao Z , Zhong Y , et al. Multiple organ infection and the pathogenesis of SARS. J Exp Med. 2005;202(3):415–24. 16043521 10.1084/jem.20050828 PMC2213088

[38] Ng O‐W , Chia A , Tan AT , Jadi RS , Leong HN , Bertoletti A , et al. Memory T cell responses targeting the SARS coronavirus persist up to 11 years post‐infection. Vaccine. 2016;34(17):2008–14. 26954467 10.1016/j.vaccine.2016.02.063 PMC7115611

[39] Alexandroff AB , Jackson AM , O'Donnell MA , James K . BCG immunotherapy of bladder cancer: 20 years on. Lancet. 1999;353(9165):1689–94. 10335805 10.1016/S0140-6736(98)07422-4

[40] Kawai K , Miyazaki J , Joraku A , Nishiyama H , Akaza H . Bacillus Calmette‐Guerin (BCG) immunotherapy for bladder cancer: current understanding and perspectives on engineered BCG vaccine. Cancer Sci. 2013;104(1):22–7. 23181987 10.1111/cas.12075 PMC7657210

[41] Zlotta AR , Drowart A , Van Vooren J‐P , de Cock M , Pirson M , Palfliet K , et al. Evolution and clinical significance of the T cell proliferative and cytokine response directed against the fibronectin binding antigen 85 complex of bacillus Calmette‐Guerin during intravesical treatment of superficial bladder cancer. J Urol. 1997;157(2):492–8. 8996341

[42] Redelman‐Sidi G , Glickman MS , Bochner BH . The mechanism of action of BCG therapy for bladder cancer—a current perspective. Nat Rev Urol. 2014;11(3):153. 24492433 10.1038/nrurol.2014.15

[43] Arts RJ , Moorlag SJ , Novakovic B , Li Y , Wang S‐Y , Oosting M , et al. BCG vaccination protects against experimental viral infection in humans through the induction of cytokines associated with trained immunity. Cell Host Microbe. 2018;23(1):89–100.e5. 29324233 10.1016/j.chom.2017.12.010

[44] Stylianou E , Paul MJ , Reljic R , McShane H . Mucosal delivery of tuberculosis vaccines: a review of current approaches and challenges. Expert Rev Vaccines. 2019;18(12):1271–84. 31876199 10.1080/14760584.2019.1692657 PMC6961305

[45] Rosenthal SR , McEnery JT , Raisys N . Aerogenic BCG vaccination against tuberculosis in animal and human subjects. J Asthma Res. 1968;5(4):309–23. 5666107 10.3109/02770906809100348

[46] Graham RL , Donaldson EF , Baric RS . A decade after SARS: strategies for controlling emerging coronaviruses. Nat Rev Microbiol. 2013;11(12):836–48. 24217413 10.1038/nrmicro3143 PMC5147543

[47] Shi J , Wen Z , Zhong G , Yang H , Wang C , Huang B , et al. Susceptibility of ferrets, cats, dogs, and other domesticated animals to SARS–coronavirus 2. Science. 2020;eabb7015. 10.1126/science.abb7015 PMC7164390 32269068

[48] Kim Y‐I , Kim S‐G , Kim S‐M , Kim E‐H , Park S‐J , Yu K‐M , et al. Infection and rapid transmission of SARS‐CoV‐2 in Ferrets. Cell host. Cell Host Microbe. 2020;27(5):704–709.e2. 32259477 10.1016/j.chom.2020.03.023 PMC7144857

[49] Ayoub B . COVID‐19 vaccination clinical trials should consider multiple doses of BCG. Pharmazie. 2020;75(4):159. 10.1691/ph.2020.0444 32295694

[50] Saif LJ . Vaccines for COVID‐19: perspectives, prospects, and challenges based on candidate SARS, MERS, and animal coronavirus vaccines. Euro Med J. 2020. 10.33590/emj/200324

